# The Impact of Autistic Traits on Social Cognitive Performance in Schizophrenia Spectrum Disorders

**DOI:** 10.1192/j.eurpsy.2025.626

**Published:** 2025-08-26

**Authors:** Y. E. Turan, B. Verim, Y. Dokuyan, A. Küçükakdağ, E. Cesim, B. Yalınçetin, E. Bora

**Affiliations:** 1 Department of Neurosciences, Dokuz Eylul University; 2 Departmant of Psychiatry, İzmir City Hospital; 3 Departmant of Psychiatry, Faculty of Medicine, Dokuz Eylul University, İzmir, Türkiye; 4 Departmant of Psychiatry, University of Melbourne and Melbourne Health, Melbourne Neuropsychiatry Centre, Carlton South, Victoria, Australia

## Abstract

**Introduction:**

Social cognition impairments are well-recognized in both Schizophrenia Spectrum Disorders (SSDs) and Autism Spectrum Disorder (ASD), significantly impacting interpersonal relationships and overall quality of life. While some studies have suggested differences in social cognition between these two disorders, recent research has shown that these differences may be non-significant when controlling for factors such as age and symptom severity(Pinkham et al. Psychol Med 2019; 1-9). Given the overlap in symptoms and the potential for autistic traits to influence social cognitive functioning in SSD, it’s crucial to investigate how these traits can impact social cognition in individuals with SSD.

**Objectives:**

The aim of this study is to evaluate autistic traits in SSD and its relation with social cognition and clinical variables.

**Methods:**

73 participants with SSD (56 patients (mean age 37.80 ± 11.519) with schizophrenia and 17 patients (mean age 36.47± 10.909) with schizoaffective disorder) participated in the study. All participants were interviewed using the Structured Clinical Interview for DSM-IV. Current psychotic, negative and positive symptoms of all patients were evaluated using the Scale for the Assessment of Positive Symptoms and the Brief Negative Symptom Scale (BNSS). The Screen for Cognitive Impairment in Psychiatry(SCIP) was used for measuring cognitive function. Autistic traits are evaluated with the Comprehensive Autism Trait Inventory (CATI). In the assessment of social cognition, facial emotion recognition was evaluated with Penn Emotion Recognition Test (PERT), theory of mind was assessed Reading Mind in The Eyes (RMET) task.

**Results:**

Total CATI scores weren’t correlated with RMET and PERT scores. Communication and social camouflage subscores of CATI were negatively correlated with total RMET score (r=-0.289, p=0.013; r=-0.265, p=0.024). CATI total/subscale scores didn’t have a relationship with age, education years, BNSS and SCIP scores. SCIP score was correlated with RMET(r=0.358, p<0.01) and PERT (r=0.259, p=0.027). Age had a negative relationship with RMET(r=-0.27, p=021) and PERT (r=-0.397, p<0.01) scores while education was positively correlated with RMET even though the the strength was low (r=0.246, p=0.036).

**Conclusions:**

Contrary to expectations, the results did not show a relationship between higher autistic traits in SSD and social cognitive performance. The social subscales of CATI revealed a negative correlation between higher autistic traits and theory of mind performance, but no correlation with emotion recognition. In accordance with literature, aging shows a relationship with lower social cognition scores. Future research should further investigate how autistic traits impact theory of mind deficits in SSD. Interventions regarding social cognitive deficits with age should be evaluated.

**Disclosure of Interest:**

None Declared

